# Reducing Cytoplasmic Polyamine Oxidase Activity in *Arabidopsis* Increases Salt and Drought Tolerance by Reducing Reactive Oxygen Species Production and Increasing Defense Gene Expression

**DOI:** 10.3389/fpls.2016.00214

**Published:** 2016-02-29

**Authors:** G. H. M. Sagor, Siyuan Zhang, Seiji Kojima, Stefan Simm, Thomas Berberich, Tomonobu Kusano

**Affiliations:** ^1^Graduate School of Life Sciences, Tohoku UniversitySendai, Japan; ^2^Frontier Research Institute for Interdisciplinary Sciences, Tohoku UniversitySendai, Japan; ^3^Department of Biosciences, Molecular Cell Biology of Plants, Goethe UniversityFrankfurt am Main, Germany; ^4^Biodiversity and Climate Research Center, Laboratory CenterFrankfurt am Main, Germany

**Keywords:** *Arabidopsis thaliana*, drought stress, loss-of-function mutant, polyamine oxidase, reactive oxygen species, salinity stress

## Abstract

The link between polyamine oxidases (PAOs), which function in polyamine catabolism, and stress responses remains elusive. Here, we address this issue using *Arabidopsis pao* mutants in which the expression of the five *PAO* genes is knocked-out or knocked-down. As the five single *pao* mutants and wild type (WT) showed similar response to salt stress, we tried to generate the mutants that have either the cytoplasmic PAO pathway (*pao1 pao5*) or the peroxisomal PAO pathway (*pao2 pao3 pao4*) silenced. However, the latter triple mutant was not obtained. Thus, in this study, we used two double mutants, *pao1 pao5* and *pao2 pao4*. Of interest, *pao1 pao5* mutant was NaCl- and drought-tolerant, whereas *pao2 pao4* showed similar sensitivity to those stresses as WT. To reveal the underlying mechanism of salt tolerance, further analyses were performed. Na uptake of the mutant (*pao1 pao5*) decreased to 75% of WT. PAO activity of the mutant was reduced to 62% of WT. The content of reactive oxygen species (ROS) such as hydrogen peroxide, a reaction product of PAO action, and superoxide anion in the mutant became 81 and 72% of the levels in WT upon salt treatment. The mutant contained 2.8-fold higher thermospermine compared to WT. Moreover, the mutant induced the genes of salt overly sensitive-, abscisic acid (ABA)-dependent- and ABA-independent- pathways more strongly than WT upon salt treatment. The results suggest that the *Arabidopsis* plant silencing cytoplasmic *PAO*s shows salinity tolerance by reducing ROS production and strongly inducing subsets of stress-responsive genes under stress conditions.

## Introduction

Polyamines (PAs) are aliphatic compounds with low molecular masses that are ubiquitously present in all living organisms. Bacteria primarily contain the PAs putrescine (Put) and spermidine (Spd), whereas some bacteria and mammalian cells also contain spermine (Spm; Tabor and Tabor, 1985; Cohen, 1998). In addition to these PAs, plants also contain another tetraamine, thermospermine (T-Spm), an isomer of Spm (Knott et al., 2007; Kakehi et al., 2008; Naka et al., 2010; Takano et al., 2012). PAs play important roles in numerous physiological processes. In plants, PAs are involved in embryogenesis, cell division, organogenesis, flowering and senescence, as well as responses to abiotic and biotic stresses (Groppa and Benavides, 2008; Kusano et al., 2008; Alcázar et al., 2010; Mattoo et al., 2010; Minocha et al., 2014; Berberich et al., 2015; Mo et al., 2015)

Polyamine homeostasis is governed by a dynamic balance between PA biosynthesis and catabolism. The plant PA biosynthetic pathway has been well documented (Bagni and Tassoni, 2001; Kusano et al., 2008; Fuell et al., 2010). Put biosynthesis starts with either the conversion of ornithine by ornithine decarboxylase (ODC) or arginine by arginine decarboxylase. Put is then converted to Spd by Spd synthase. This reaction requires another substrate, decarboxylated *S*-adenosylmethionine, which is synthesized from methionine via two sequential reactions catalyzed by methionine adenosyltransferase and *S*-adenosylmethionine decarboxylase, respectively. Spd is further converted to either Spm or T-Spm, which is catalyzed by Spm synthase or T-Spm synthase, respectively. It should be noted that *Arabidopsis thaliana* lacks a gene encoding ODC (Hanfrey et al., 2001). Accumulating evidence indicates that transgenic plants with increased PA levels (via overexpression of PA biosynthetic genes) have increased abiotic stress tolerance, whereas mutant plants deficient in PA biosynthesis are hypersensitive to abiotic stresses (Urano et al., 2003; Capell et al., 2004; Yamaguchi et al., 2006, 2007; Berberich et al., 2015 and the references therein).

Two additional enzymes, copper-containing amine oxidase (CuAO) and polyamine oxidase (PAO), are also involved in PA catabolism (Bagni and Tassoni, 2001; Cona et al., 2006; Angelini et al., 2008; Moschou et al., 2012; Kusano et al., 2015; Liu et al., 2015; Wang and Liu, 2015). It was previously thought that CuAO only catalyzed the oxidation of diamines. For example, Put is oxidized to 4-aminobutanal with concurrent production of NH_3_ and H_2_O_2_. However, it was recently revealed that some CuAOs also oxidize the triamine Spd (Planas-Portell et al., 2013). PAO is a flavin-adenine dinucleotide (FAD)-associated enzyme. Until 2006, it was believed that plant PAO catalyzed the conversion of Spd- and Spm-oxidation to 4-aminobutanal and *N*-(3-aminopropyl)-4-aminobutanal, respectively, along with the production of 1,3-diaminopropane and H_2_O_2_ (Federico et al., 1990; Cona et al., 2006). The mode of this reaction is known as terminal catabolism. In addition to this process, plant PAO is also involved in a PA back-conversion pathway (Tavladoraki et al., 2006; Kamada-Nobusada et al., 2008; Moschou et al., 2008c; Takahashi et al., 2010; Fincato et al., 2011, 2012). This type of PAO reaction converts Spm and T-Spm to Spd, and/or further to Put, along with the production of 3-aminopropanal and H_2_O_2_. *A. thaliana* contains five *PAO* genes, termed *AtPAO1* to *AtPAO5*. These genes and their products have been extensively studied. AtPAO1 and AtPAO5 are located in the cytoplasm, whereas AtPAO2, AtPAO3, and AtPAO4 reside in peroxisomes (Tavladoraki et al., 2006; Kamada-Nobusada et al., 2008; Moschou et al., 2008c; Takahashi et al., 2010; Fincato et al., 2011, 2012; Ahou et al., 2014; Kim et al., 2014). Although the five AtPAOs differ in their spatio-temporal expression patterns and PA substrate specificity, all of these PAOs are involved in PA back-conversion, namely, AtPAO1 and AtPAO5 prefer T-spm and back-convert it to Spd, AtPAO4 is involved in Spm back-conversion to Spd, not to Put, and AtPAO2 and AtPAO3 mainly convert Spd to Put. While a link between PA catabolism and abiotic and biotic stress responses has been described (Berberich et al., 2015; Roach et al., 2015, and references therein), most of these results were obtained using CuAO- or PAO-specific inhibitors.

The aim of this study was to uncover distinct role(s) of *Arabidopsis* PAOs in abiotic stress responses. We examined the growth responses of knock-out or knock-down mutants of *AtPAO*s under high salt and drought stress conditions. We found that the *pao1 pao5* double mutant, but not the five single mutants or the *pao2 pao4* double mutant, was tolerant to salt and drought stress. We investigated the reason behind the salt tolerance of *pao1 pao5*, finding that several genes in ABA-dependent and -independent pathways were highly expressed in this mutant. We also measured the PAO activity and the amounts of hydrogen peroxide (H_2_O_2_) and superoxide anion (O_2_^-^) in the *pao1 pao5* mutant. The results of this study help elucidate a possible mechanism underlying the salt and drought tolerance of *pao1 pao5*.

## Materials and Methods

### Plant Materials and Growth Conditions

*Arabidopsis thaliana* accession Col-0 [wild type (WT)] and T-DNA insertion lines *Atpao1-2* (SAIL_822_A11), *Atpao2-4* (SALK_046281), *Atpao3-1* (GK209F07), *Atpao4-1* (SALK_133599), and *Atpao5-2* (SALK_053110), which were obtained from the *Arabidopsis* Biological Resource Center (Ohio State University, USA), were used in this study. The T-DNA insertion lines were designated *pao1*, *pao2*, *pao3*, *pao4*, and *pao5*, respectively. All seeds were surface sterilized with 70% ethanol for 1 min and 1% sodium hypochloride plus 0.1% Tween-20 for 15 min, followed by extensive washing with sterile distilled water. Sterilized seeds were placed into pots containing a soil mix consisting of Vermiculite:SupermixA (1:1 v/v) or on half-strength MS (designated 1/2 MS in this study) 1.5% agar plates (pH 5.6) containing 1% sucrose and B5 vitamin (MP Biomedicals, Cat # 2625149). The plants were grown at 22°C under a 14 h light/10 h dark photocycle.

### Chemicals

Put, Spd, and Spm were purchased from Tokyo Kasei Co., Ltd. (Tokyo, Japan). T-Spm was chemically synthesized (Niitsu and Samejima, 1986). All other chemicals, which were analytical grade, were purchased from Sigma–Aldrich Corp. (St. Louis, MO, USA), Wako Pure Chemical Industries, Ltd. (Osaka, Japan), and Nacalai Tesque, Ltd. (Kyoto, Japan).

### Reverse Transcription-PCR (RT-PCR) and Quantitative RT-PCR (qRT-PCR) Analyses

Total RNA was prepared from 2-week-old *Arabidopsis* seedlings using Sepasol-RNA I Super (Nacalai Tesque, Kyoto, Japan). First-strand cDNA was synthesized with ReverTra Ace (Toyobo Co. Ltd., Osaka, Japan) and oligo-dT primers. The qRT-PCR analysis was performed with Fast-Start Universal SYBR Green Master (ROX; Roche Applied Science, Mannheim, Germany) on a StepOne real-time PCR system (Life Technologies Japan, Tokyo, Japan). The two-step RT-PCR was performed with the following program: one cycle of 95°C for 10 min, followed by 40 cycles of 95°C for 15 s, and 60°C for 60 s. Melting curves were generated after the 40 cycles by heating the samples to 95°C for 15 s, followed by cooling to 60°C for 1 min and heating to 95°C for 15 s. The amount of cDNA was calculated with the comparative ΔΔ*C*_T_ method (Schmittgen and Livak, 2008) with StepOne v2.1 (Applied Biosystems) using the housekeeping gene *CBP20*, encoding cap-binding protein 20 (Supplementary Table S2) as a reference gene.

### Growth Response to High Salt and Drought Treatment

High salt treatment: Sterilized *Arabidopsis* seeds were grown on 1/2 MS agar plates containing different concentrations of NaCl (0, 25, 50, 75, and 100 mM). The plates were placed at a vertical position with an 85° angle and incubated in a growth chamber at 22°C for 14 days. Drought treatment: *Arabidopsis* seeds were sown in pots containing soil mix (Vermiculite: Supermix A, 1:1 v/v) in a plant incubator at 22°C under a 14 h light/10 h dark photocycle. Each pot contained 28 g of soil mix. The plants were supplied with 50 ml of water once a week for 1 month. The plants were then divided into two groups: the first group was grown as before, and the second group was subjected to drought stress by withholding water for 2 weeks.

### Generation of the AtPAO Double Mutants

The *pao1 pao5* and *pao2 pao4* double mutant plants were generated by crossing *pao1* with *pao5*, and *pao2* with *pao4*, respectively.

### Water Loss Assay

Water loss assay was performed by the procedure described by Weigel and Glazebrook (2002). In brief, 2-week-old *Arabidopsis* seedlings were removed from the 1/2 MS agar plates, and 25 seedlings per plate were placed onto dry filter paper. Their fresh weights were monitored every 10 min for 60 min after the onset of drought treatment. The fresh weights at the onset of the treatment were set at 100%, and the relative water loss was determined.

### Measuring Na and K Levels

Two-week-old seedlings grown on 1/2 MS agar medium were carefully removed from the plates, transferred to wet filter paper containing 1/2 MS liquid medium with or without 100 mM NaCl, and further incubated for 12 and 24 h, respectively. The plant samples were collected, rinsed three times with deionized water, and dried at 65°C for 2 days. Dried plant samples were digested with 100% nitric acid at 130°C for 90 min and filtered, and the ion concentrations in the samples were analyzed by ICP spectrophotometry (iCAP 6000 series, ThermoFisher Scientific Inc., Waltham, MA, USA).

### PAO Activity Assay

Polyamine oxidases activity was assayed as described by Liu and Liu (2004). Briefly, the enzyme extracts were prepared as follows: 2-week-old seedlings (approximately 0.2 g) were homogenized in 100 mM phosphate buffer (pH 8.0) containing 20 mM sodium ascorbate, 1 mM pyridoxal-5′-phosphate, 10 mM DTT, 0.1 mM Na_2_EDTA, and 0.1 mM PMSF (phenylmethylsulfonyl fluoride). The homogenate was centrifuged at 15,000 × *g* for 60 min at 4°C. The supernatant was used as the crude enzyme extract. The reaction mixture (total volume of 3.0 mL) contained 0.1 mL of crude enzyme extract, 2.5 mL of 100 mM sodium phosphate buffer (pH 6.5), 0.2 ml of 4-aminoantipyrine/*N,N*′-dimethylaniline, and 0.1 mL of horseradish peroxidase (POX; 250 U/mL). The reaction was initiated by adding 200 mM spermidine. A 0.01 change in absorbance at 555 nm was regarded as one enzyme activity unit.

### ROS Analyses

H_2_O_2_ levels were determined as described by Messner and Boll (1994). Seedlings (approximately 200 mg fresh weight) were homogenized in a pre-chilled mortar and pestle with 0.3 mL of 100 mM potassium phosphate buffer (pH 7.0) containing 10% (w/v) Polyclar. Each aliquot (200 μL) of extract was combined with 10 μL of horseradish POX [1 mg of enzyme (169-10791, Wako Chemical Co. Ltd.) dissolved in 100 mM potassium phosphate buffer, 100 units/mg] and 10 μL of 50 mM (w/v) ABTS [2,20-azino-bis(3-ethylobenzo-thiazoline-6-sulfonic acid) diammonium salt] solution. After 3 min of incubation, absorbance at 415 nm was measured using a spectrophotometer and compared with the values obtained from standard solutions containing specific amounts of H_2_O_2_ in 100 mM potassium phosphate buffer (pH 7.0).

O_2_^-^ levels were determined based on its ability to reduce nitro blue tetrazolium (NBT) as described (Doke, 1983). In brief, seedlings were cut into pieces and immersed in 10 mM potassium phosphate buffer (pH 7.8) containing 0.05% (w/v) NBT and 10 mM NaN_3_, and incubated for 1 h at room temperature. After incubation, 2 mL of the reaction solution was heated at 85°C for 15 min and cooled rapidly. The optical density at 580 nm was recorded, and the O_2_^-^ content was expressed as the increase in absorbance/g dry weight.

*In situ* accumulation of O_2_^-^ and H_2_O_2_ was detected by histochemical staining with NBT and diaminobenzidine (DAB), respectively, according to the procedure described by Romero-Puertas et al. (2004) with minor modifications. In brief, 2-week-old *Arabidopsis* seedlings grown on 1/2 MS agar medium were carefully removed from the medium, transferred to wet filter paper containing 1/2 MS medium solution either with or without 100 mM NaCl, and further incubated for 12 h. For O_2_^-^ detection, the seedlings were immersed in 2% NBT solution dissolved in 10 mM phosphate buffer (pH 7.8) at room temperature. The immersed leaves were illuminated for 2 h until dark spots appeared, which are characteristic of blue formazan precipitates. To detect H_2_O_2_, another set of samples was immersed in DAB solution (1 mg mL^-1^) that was freshly prepared in 10 mM phosphate buffer (pH 7.8) and incubated at room temperature for 8 h under continuous light until brown spots, which are derived from the reaction of DAB with H_2_O_2_, were observed. For both staining methods, the seedlings were bleached in 100% ethanol, followed by 70% ethanol, and observed under a microscope (LG-PS2, Olympus).

### Ion Leakage Assay

Conductivity was measured with a MultiLine Multi 3410 IDS instrument equipped with a TetraCon 925 conductivity cell (WTW Wissenschaftlich-Technische Werkstätten GmbH, Weilheim, Germany). Five seedlings each with or without 100 mM NaCl treatment were rinsed five times with distilled water. The rosette leaves removed from seedlings were incubated in a 15 mL tube with 5 mL of distilled water for 24 h at 22°C before measuring (Cond1). Total conductivity (Cond2) was obtained after heating the tubes in boiling water for 20 min. Electrolyte leakage was expressed as percentage of total conductivity. Leakage was calculated as: Cond1/Cond2 × 100.

### Enzyme Activity Assay

The activities of catalase (CAT; EC 1.11.1.6), POX (EC 1.11.1.7), superoxide dismutase (SOD; EC 1.15.1.1) and ascorbate peroxidase (APX; EC 1.10.3.3) were tested in 100 mM NaCl treated and untreated WT and *pao1 pao5* mutant plants. Five seedlings each were blotted dry, weighed and homogenized on ice in the respective extraction buffer (4 μL/mg fresh weight) in a 1.5 mL tube with a little sea sand and a pestle. Fifty mM K-phosphate-buffer, pH 7.5, was used as extraction buffer for CAT and SOD. For APX assays 50 mM K-phosphate-buffer, pH 7.0, containing 1 mM Na-ascorbate was used for extraction. For POX assays the assay buffer of the assay Kit (Sigma–Aldrich) was used. After centrifugation at 15,000 × *g*, for 10 min at 4°C, the supernatants were transferred to fresh tubes and kept on ice until use. All enzyme activities were measured spectrophotometrically (Hitachi U-2000). The results are presented as relative activities compared to untreated WT seedlings. CAT activity was estimated by the breakdown of H_2_O_2_ (Beers and Sizer, 1952). Briefly, 10 μL of extract was used in a total volume of 1 mL extraction buffer containing 10 mM H_2_O_2_ and the decrease of H_2_O_2_ was monitored by change in the absorbance at 240 nm. The activity per mg tissue was calculated according to Beers and Sizer (1952). POX activity was measured using a commercial kit (POX Activity Assay Kit, Sigma–Aldrich). Four μL of extract was used for the colorimetric assay (570 nm) in a total volume of 100 μL as described in the manual using a microcuvette at 25°C. A standard curve was used to calculate enzyme activity per mg tissue. SOD was assayed according to McCord and Fridovich (1969) through inhibition of the reduction of oxidized cytochrome c by SOD in a coupled system, using xanthine and xanthine oxidase. Between 5 and 10 μL of extract were used and the absorbance at 550 nm was monitored. The percentage of inhibition was calculated and used to express the activity in units/g FW. APX activity was tested with 10 μL of extract in a total volume of 1 mL reaction mixture by monitoring the oxidation rate of ascorbate at 290 nm according to Nakano and Asada (1987).

### PA Analysis by High Performance Liquid Chromatography (HPLC)

PA extraction, derivatization, and analysis by HPLC were performed as described by Naka et al. (2010) and Sagor et al. (2015). In brief, plant samples (0.3–0.5 g per sample) were pulverized with a mortar and pestle under liquid nitrogen. Five volumes (2.5 mL per 0.5 g of plant sample) of 5% (v/v) cold perchloric acid were added to the resulting fine powders. The mixtures were transferred to plastic tubes and kept on ice for 1 h. After centrifugation at 15,000 × *g* for 30 min at 4°C, the supernatants were combined and filtered using a filter syringe (pore size, 0.2 μm). One milliliter of 2 N NaOH was added to 1.5 mL of plant extract, the mixture was vortexed, 10 μL of benzoyl chloride was added, and mixed again followed by incubation at room temperature for 20 min, and then 2 mL of saturated NaCl was added. After the addition of 2 mL of diethyl ether, samples were vigorously mixed and then centrifuged at 3,000 × *g* for 10 min at 4°C for phase-separation. An aliquot (1.5 mL) of the organic solvent phase was evaporated and the residue was resuspended in 50 μL of methanol.

Benzoylated PAs were analyzed with a programmable Agilent 1200 liquid chromatograph using a reverse-phase column (4.6 mm × 250 mm, TSK-GEL ODS-80Ts, TOSOH, Tokyo, Japan) and detected at 254 nm as described (Sagor et al., 2015).

### Statistical Analysis

All experiments were performed with at least three biological samples unless mentioned. Data analysis was performed using the statistical tools (Student’s *t*-test) of Microsoft Excel software.

## Results

### Five *Arabidopsis* Single *pao* Mutants Exhibit WT-Like Responses to High Salt Stress

First, we confirmed the transcript levels of the five *PAO* genes in WT and the *pao* mutants by real-time reverse transcription-polymerase chain reaction (qRT-PCR) using the primers listed in Supplementary Table S1. The *pao1* and *pao5* mutants appeared to be knock-out mutants, whereas *pao2*, *pao3*, and *pao4* were likely knock-down mutants (Kim et al., 2014). We tested the NaCl sensitivity of these *pao* mutants by plating sterilized seeds on MS agar medium with or without 100 mM NaCl and incubating them for 14 days. Under non-stressed conditions, the relative growth of the *pao* mutants was similar to WT, and no difference in growth was observed among mutants (Supplementary Figure S1A). When grown on 100 mM NaCl, primary root growth was inhibited to a similar extent among WT and all *pao* mutants (Supplementary Figures S1A,B).

### Growth Responses of *pao1pao5* and *pao2pao4* Mutants Under High Salt Stress

**FIGURE 1:**
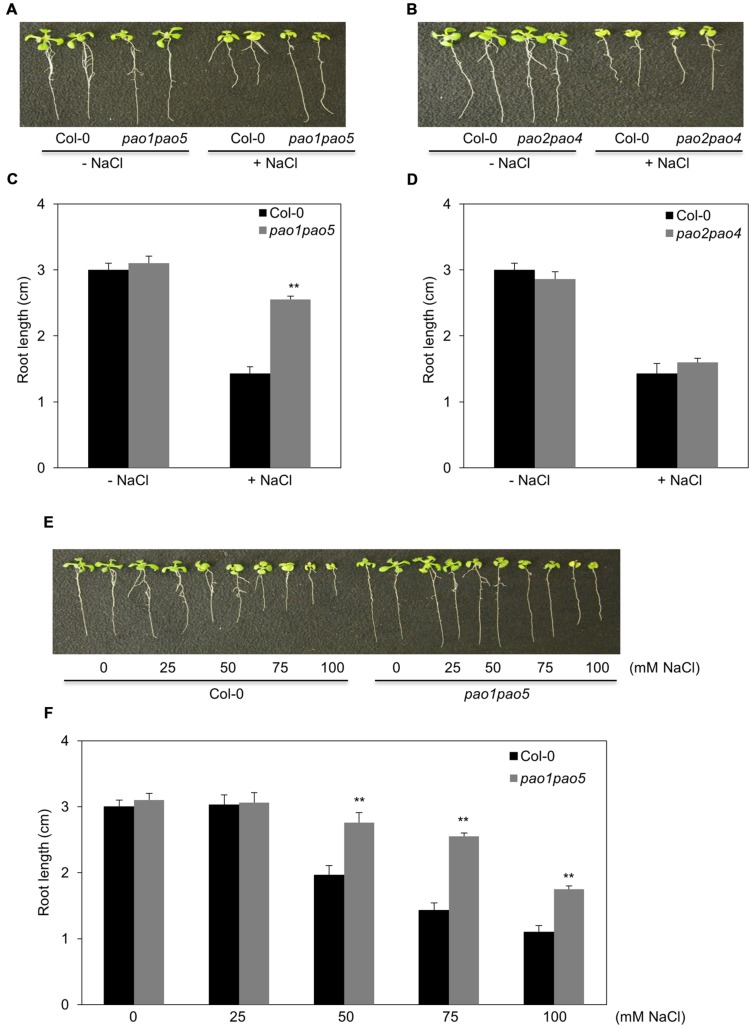
**Growth responses of *pao1 pao5* and *pao2 pao4* to various concentrations of NaCl. (A)** Growth response of *pao1 pao5* to 100 mM NaCl. **(B)** Root lengths of WT and *pao1 pao5* with or without 100 mM NaCl after a 14 days incubation. The values indicate means ± SE (*n* = 5). **(C)** Growth response of *pao2 pao4* to 100 mM NaCl. **(D)** Average root length of WT and *pao2 pao4* with or without 100 mM NaCl after a 14 days incubation. The values indicate means ± SE (*n* = 5). **(E)** Growth responses of WT and *pao1pao5* plants to different NaCl levels in MS agar medium. Two representative plants, each incubated for 14 days, were photographed. **(F)** Root length of WT and *pao1 pao5* grown on MS agar medium containing 0, 25, 50, 75, and 100 mM NaCl for 14 days. The values indicate means ± SE (*n* = 5). Asterisk indicates significant difference between WT (Col-0) and *pao1 pao5*; ^∗∗^*P* < 0.01.

Of the five AtPAOs, two (AtPAO1 and AtPAO5) localize to the cytoplasm and three (AtPAO2, AtPAO3, and AtPAO4) reside in peroxisome. Thus, we first aimed to generate the mutants which either lost cytoplasmic PAO activity (*pao1 pao5*) or lost peroxisomal PAO activity (*pao2 pao3 pao4*) (Andronis et al., 2014). We obtained two *pao* double mutants, *pao1 pao5* and *pao2 pao4*, by crossing. We further tried to get a triple mutant, *pao2 pao3 pao4*, by crossing *pao2 pao4* and *pao3*, three times, but failed to get it. Therefore, we used the two double mutants, *pao1 pao5* and *pao2 pao4*, in this study. We confirmed the identity of the mutants by PCR, finding that both double mutants contained T-DNA inserted in both DNA strands of *PAO1* and *PAO5* and of *PAO2* and *PAO4*, respectively (Supplementary Figure S2). We then examined *PAO* transcript levels in the mutants. In *pao1pao5*, *AtPAO2*, *AtPAO3*, and *AtPAO4* transcript levels were a bit higher than those of WT, whereas *AtPAO1* and *AtPAO5* transcript levels were reduced to 20 and 0.7%, respectively, compared to WT (Supplementary Figures S3A,B). In *pao2 pao4*, the levels of *AtPAO1*, *AtPAO2*, *AtPAO3*, *AtPAO4*, and *AtPAO5* transcripts were approximately 150, 0.03, 170, 18, and 160% those of WT, respectively (Supplementary Figures S3A,C), further confirming the identity of both mutants. Next, we tested the growth of these double mutants under high salt conditions. Interestingly, *pao1 pao5* was less sensitive to NaCl than WT (**Figures 1A,B**), whereas *pao2 pao4* showed WT levels of NaCl sensitivity (**Figures 1C,D**). The use of increasing concentrations of NaCl confirmed the NaCl tolerance of *pao1 pao5*. Finally, the primary roots of the mutant were significantly longer (140∼178%) than those of WT when grown in 50–100 mM NaCl (**Figures 1E,F**). Therefore, the *pao1 pao5* mutant is NaCl tolerant compared to WT.

### Growth Responses of *pao1pao5* to Drought Stress

**FIGURE 2:**
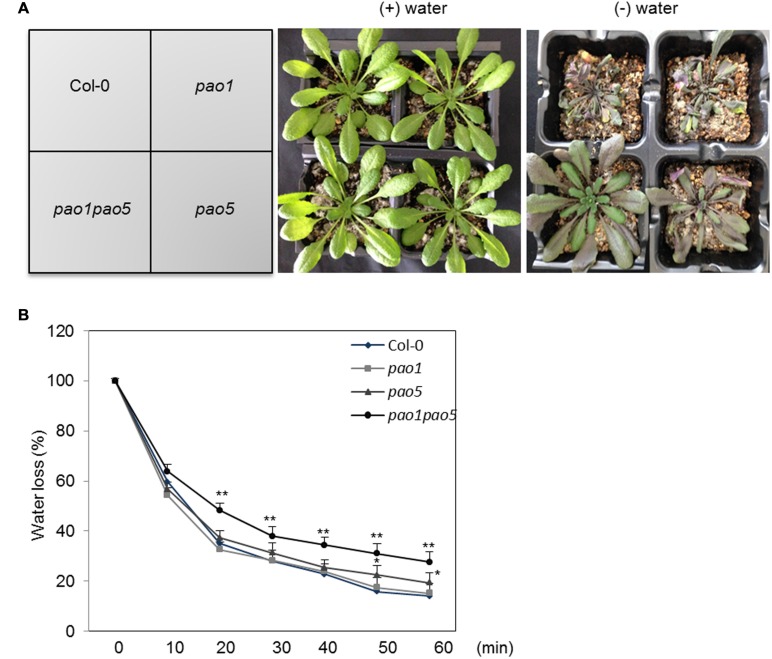
**Drought responses of WT, *pao1*, *pao5*, and *pao1 pao5* plants, and their rates of water loss. (A)** Left, WT, *pao1*, *pao5*, and *pao1 pao5* plants were grown in Vermiculite/Supermix A (1:1 v/v) for 28 days. Right, water supply was stopped at 28 days and the plants were further incubated for 14 days. **(B)** Water loss rate from WT, *pao1*, *pao5*, and *pao1 pao5* plantlets. Two-week-old plantlets (*n* = 25) grown on MS agar medium were carefully detached from the plates and placed onto dry filter paper (Whatman #2) and their fresh weight was measured at various time intervals. Asterisk indicates significant difference: ^∗^*P* < 0.05, ^∗∗^*P* < 0.01.

We also tested the growth responses of the *pao1 pao5* mutant to drought stress, along with those of WT, *pao1*, and *pao5*. Under well-watered conditions, WT, *pao1*, *pao5*, and *pao1 pao5* grew at comparable rates. By contrast, once the water supply was stopped, the double mutant lived longer than WT and both single mutants (**Figure 2A**, Supplementary Figure S4). Next we measured water loss in these plants upon dehydration. At 20 min after the onset of dehydration, the double mutant still retained approximately 50% of its water content, whereas WT and the single mutants lost more than 60% of their water contents (**Figure 2B**).

### Na and K (Potassium) Contents in WT, *pao1*, *pao5*, and *pao1pao5* Plants Exposed to High Salt

**FIGURE 3:**
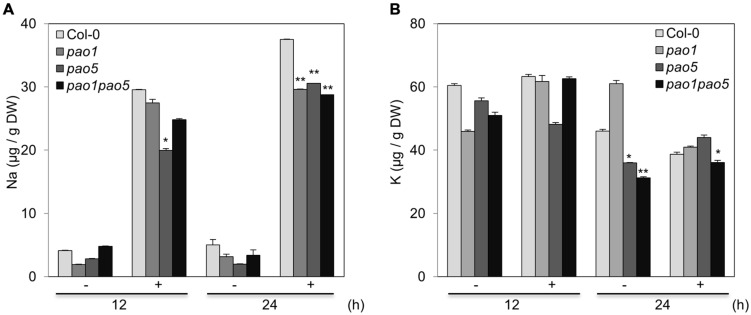
**Na (A) and K (B) contents in WT, *pao1, pao5*, and *pao1 pao5* plants exposed to 100 mM NaCl.** Asterisk indicates significant difference: ^∗^*P* < 0.05, ^∗∗^*P* < 0.01.

At 12 h after NaCl treatment, the Na and K contents in *pao5* mutant were about 70 and 80% levels compared to those of WT, whereas those in *pao1 pao5* double mutant were comparable to those of WT (**Figures 3A,B**). At 24 h of stress treatment, the Na contents in *pao1*, *pao5*, and *pao1 pao5* mutants were similarly lower (74–79%) than in WT (**Figure 3A**), whereas the K contents were similar among all plant samples (**Figure 3B**).

### Changes in PA Contents in WT and *pao1pao5* Plants Exposed to High Salt

In WT, Put levels increased to 180% at 6 h after 100 mM NaCl treatment, and then gradually decreased until 24 h but still retained 145% level. In the *pao1 pao5* mutant, Put content remained at constant levels (6 nmol/g FW) until 12 h after NaCl treatment and then increased to ca. 9 nmol/g FW at 24 h (Supplementary Figure S5A). Spd contents gradually decreased to around 75% levels in both WT and the mutant upon high salt exposure, with not much difference observed between lines (Supplementary Figure S5B). T-Spm content in *pao1 pao5* was 2.8-folds higher than that of WT under physiological condition (Supplementary Figure S5C). T-Spm content decreased to one-third level in WT at 24 h after NaCl treatment. Even in the double mutant, the T-Spm level decreased to 80% at 24 h after NaCl treatment but was still 6.5-folds higher than that of WT (Supplementary Figure S5C). Spm contents in both WT and *pao1 pao5* increased 170 and 150% levels, respectively, at 24 h after salt treatment, although their modulation patterns differed (Supplementary Figure S5D).

### Comparison of PAO Activity, H_2_O_2_ Levels, and Super Oxide Anion Levels in WT vs. *pao1*, *pao5*, and *pao1pao5* Plants Exposed to High Salt

**FIGURE 4:**
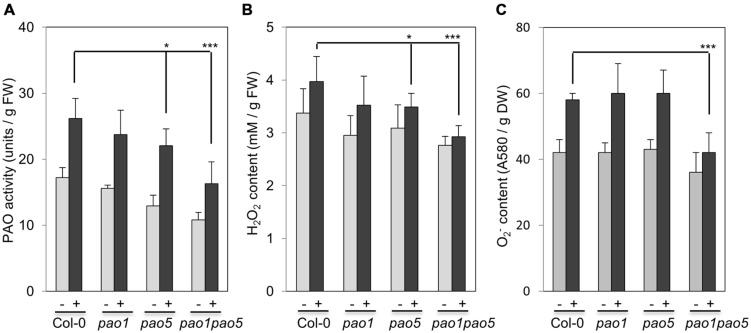
**Comparison of PAO activity (A), H_2_O_2_ production (B), and O_2_^-^ production (C) in WT, *pao1*, *pao5* single mutants, and *pao1 pao5* double mutant upon NaCl treatment.** Asterisk indicates significant difference: ^∗^*P* < 0.05, ^∗∗∗^*P* < 0.001.

**FIGURE 5:**
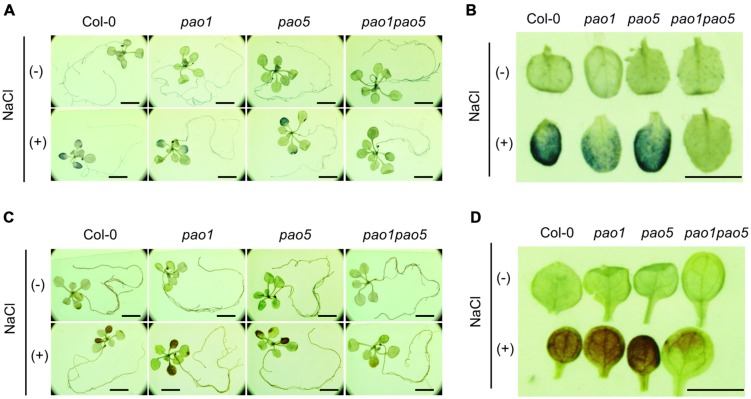
**Histochemical analysis of O_2_^-^ (A,B) and H_2_O_2_ (C,D) accumulation in WT, *pao1*, *pao5*, and *pao1 pao5* after NaCl treatment. (A,B)** NBT staining; **(C,D)** DAB staining. **(A,C)** Stained whole seedling; **(B,D)** enlarged cotyledon leaves. Bar indicates 1 mm.

We then measured PAO activity in the plants, finding that under non-stressed conditions, the highest to lowest relative PAO activity occurred in WT > *pao1* > *pao5* > *pao1 pao5* (**Figure 4A**). Under NaCl treatment, PAO activity in WT increased approximately 1.5-fold. After NaCl treatment, the plants with the highest to lowest relative PAO activity were again WT > *pao1* > *pao5* > *pao1 pao5* (**Figure 4A**). The relative PAO activity of the double mutant decreased to 62% of that of WT after NaCl treatment (**Figure 4A**). Since H_2_O_2_ is produced through the action of PAO, we examined H_2_O_2_ levels in plants after NaCl treatment. The plants with the highest to lowest H_2_O_2_ levels were WT > *pao1* = *pao5* > *pao1 pao5* under physiological- and high salt conditions. H_2_O_2_ content in the double mutant was 81% of that of WT in normal condition (**Figure 4B**). H_2_O_2_ levels in WT, *pao1* and *pao5* increased about 16–21% after NaCl treatment, whereas that in the double mutant increased 8% upon salt treatment (**Figure 4B**). We also examined the super oxide anion (O_2_^-^) levels in these plants. Under control conditions, O_2_^-^ levels were slightly lower (86%) in *pao1 pao5* than in WT and the single mutants. Upon NaCl treatment, O_2_^-^ levels increased approximately 1.4-fold in WT and in both single mutants, whereas in the double mutant, O_2_^-^ level increased to1.2-fold (**Figure 4C**). Thirty % of O_2_^-^ production decreased in the NaCl-treated double mutant compared to that in NaCl-treated WT, *pao1* and *pao5* mutants (**Figure 4C**). Collectively, the results indicate that the increased H_2_O_2_ and O_2_^-^ levels in the NaCl-treated double mutant were less significant in relative to those in NaCl-treated WT and the single mutants. The results were further supported by histochemical detection of O_2_^-^ and H_2_O_2_ (**Figure 5**). In WT, *pao1*, and *pao5* single mutants, intense blue formazan precipitates were detected after NaCl treatment, while such signals were weak in the *pao1 pao5* double mutant, which is indicative of less O_2_^-^ accumulation (**Figures 5A,B**). Similarly, intense brown precipitates were observed in WT and both single mutants treated with NaCl, whereas those in the double mutant were faint, indicating lower H_2_O_2_ accumulation in the double mutant (**Figures 5C,D**). The *pao2 pao4* mutant seemed to produce the similar levels of O_2_^-^ and H_2_O_2_ compared to those of WT upon NaCl treatment (Supplementary Figures S6A,B).

### Changes of Electrolyte Leakage and Antioxidant Enzyme Activities, and Expressional Changes of Antioxidant Enzyme Genes in WT and *pao1 pao5* Mutant

**FIGURE 6:**
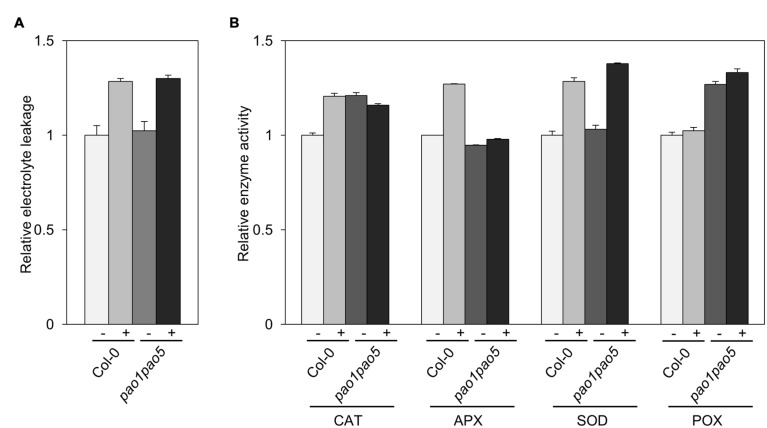
**Biochemical analyses of WT and *pao1 pao5* mutant upon high salt stress. (A)** Electrolyte leakage; **(B)** Enzyme activities of catalase (CAT), ascorbate peroxidase (APX), superoxide dismutase (SOD), and peroxidase (POX) in WT and the double mutant with or without 100 mM NaCl treatment for 6 h.

We compared the leakage rate of electrolytes between WT and the double mutant upon NaCl treatment but could not see a clear difference. Upon NaCl treatment, both WT and the mutant leaked the electrolytes ∼30% more compared to the non-treated seedlings (**Figure 6A**). Next we compared the activities of antioxidant enzymes, catalase (CAT), ascorbate peroxidase (APX), SOD and POX of WT and the mutant. The activities of CAT, APX, and SOD were enhanced ∼20–30% in WT upon NaCl treatment, while the POX activity was not changed in WT (**Figure 6B**). In the mutant, CAT and POX activities were ∼20–30% higher than those of non-stressed WT, but not modulated by salt treatment. Net SOD activity in the mutant increased to 140% levels upon salt treatment as like WT (**Figure 6B**).

**FIGURE 7:**
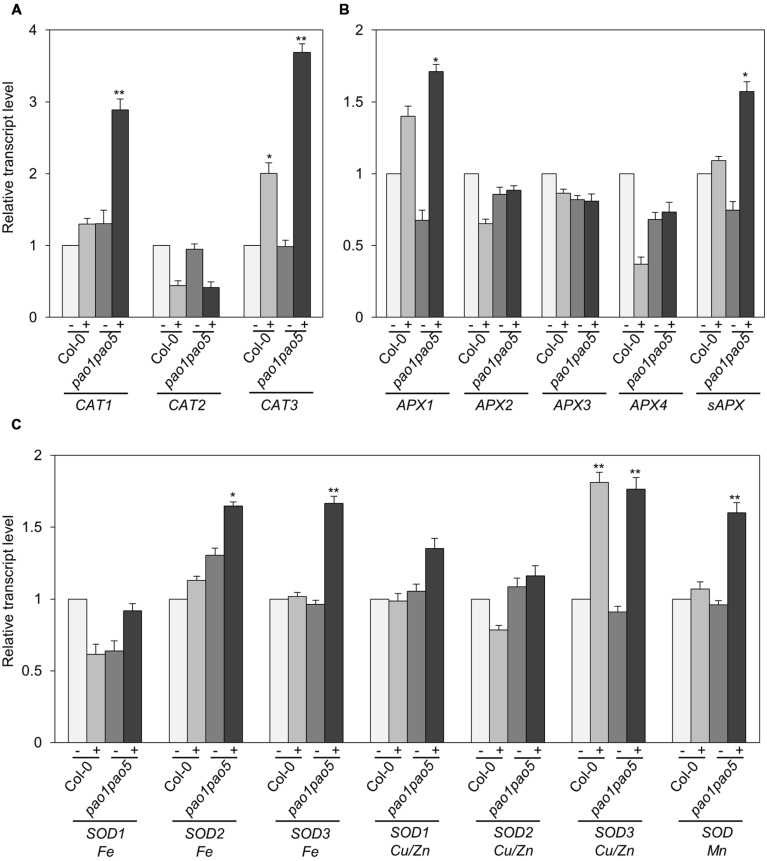
**Expressional changes of *CAT*, *APX*, and *SOD* genes in WT and *pao1 pao5* mutant upon high salt stress. (A)** *CAT* genes; **(B)** *APX* genes; **(C)** *SOD* genes. (-) control; (+) treated with 100 mM NaCl for 6 h. Asterisk indicates significant difference: ^∗^*P* < 0.05, ^∗∗^*P* < 0.01.

Furthermore we compared the transcript levels of *CAT*, *APX*, and *SOD* genes in WT and the mutant. Among three *CAT* genes, *CAT1* and CAT3 were induced 0.2-fold and 2-fold levels in WT by salt treatment, whereas they were induced 2.9 and 3.7-fold levels in the double mutant. *CAT2* transcripts decreased to ∼50% levels both in WT and the mutant after 6 h salt treatment (**Figure 7A**). Of ascorbate peroxidase genes (*APX1* to *-4*), the transcript levels of *APX2*, *APX3*, and *APX4* kept constant or decreased to 50% levels, respectively (**Figure 7B**). *APX1* and stromal ascorbate peroxidase gene (*sAPX*) behaved similarly. Namely upon salt stress *APX1* and *sAPX* transcripts increased to 1.4 and 1.2-fold, respectively, in WT, and their basal transcript levels in the double mutant were 70 and 80% compared to those of WT and they accumulated to 170 and 160% levels in relative to those of non-stressed WT (**Figure 7B**). Of three plastidial Fe-SOD genes, *Fe SOD2* and *Fe SOD3* transcripts increased to 1.6-fold levels in the double mutant (**Figure 7C**). Mitochondrial *Mn SOD* was induced to 1.6-fold levels only in the mutant after salt treatment (**Figure 7C**). *Cu/Zn SOD1* and *Cu/Zn SOD2* were not much modulated at the transcriptional level by salt treatment, whereas *Cu/Zn SOD3* was induced to 1.8-fold levels both in WT and the mutant (**Figure 7C**).

### Expressional Changes of Key Genes Involved in Adaptation to High Salt Stress in WT and *pao1 pao5* Mutant

**FIGURE 8:**
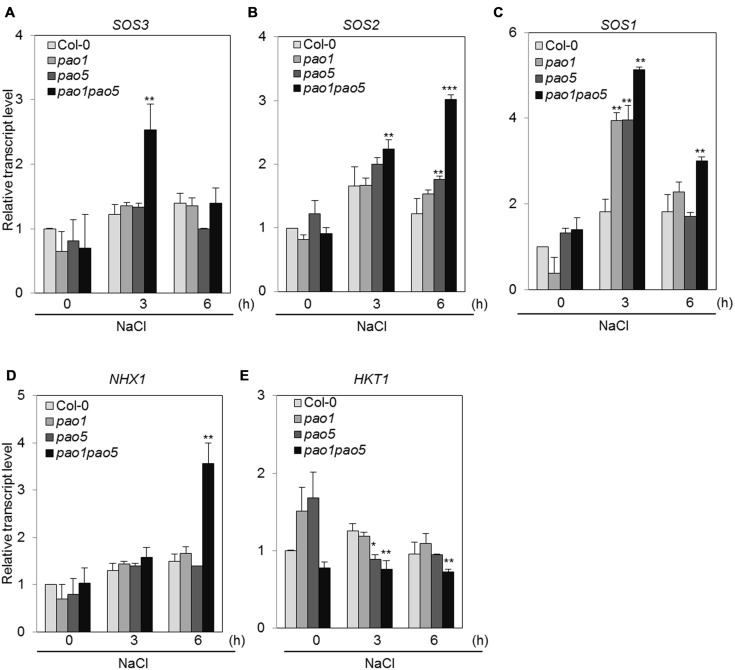
**Changes in the expression of genes involved in the SOS-signaling pathway (A–C) and Na transporter genes (D,E) in WT, *pao1*, *pao5*, and *pao1 pao5* upon high salt stress.** Asterisk indicates significant difference: ^∗^*P* < 0.05, ^∗∗^*P* < 0.01, ^∗∗∗^*P* < 0.001.

Previous investigations have revealed several genes encoding key components involved in adaptation to high salt conditions in *Arabidopsis* (Zhu, 2002). One such pathway is the Ca^2+^-dependent Salt Overly Sensitive (SOS) pathway, which consists of three components: SOS3 (calcium binding protein), SOS2 (SNF1-like protein kinase), and SOS1 (plasma membrane-localized Na^+^/H^+^ antiporter; Mahajan et al., 2008; Ji et al., 2013). In addition, HKT1 (high affinity potassium transporter1 localized in plasma membrane) and NHX1 (vacuolar-localized Na^+^/H^+^ exchanger) play important roles in Na^+^ uptake and Na^+^-sequestration to vacuole, respectively (Apse et al., 1999). We therefore measured the transcript levels of genes encoding these key components. Among SOS pathway genes, *SOS3* was transiently upregulated to twofold levels in the double mutant after 3 h NaCl treatment and returned to the basal level at 6 h, while *SOS2* transcripts gradually accumulated to threefold levels in the double mutant compared to WT at 6 h (**Figures 8A,B**). *SOS1* transcripts accumulated to 1.8-fold in WT both at 3 and 6 h after NaCl treatment (**Figure 8C**). In both the single mutants, they accumulated to fourfold levels at 3 h after NaCl treatment and returned to twofold levels as similar as WT (**Figure 8C**). In the double mutant, they reached to five and threefold levels, respectively, at 3 and 6 h compared to those of non-stressed WT (**Figure 8C**). Upregulation of *NHX1* occurred at 6 h after NaCl treatment only in the double mutant, whereas no change in *HKT1* expression was observed in any plants after NaCl treatment (**Figures 8D,E**).

**FIGURE 9:**
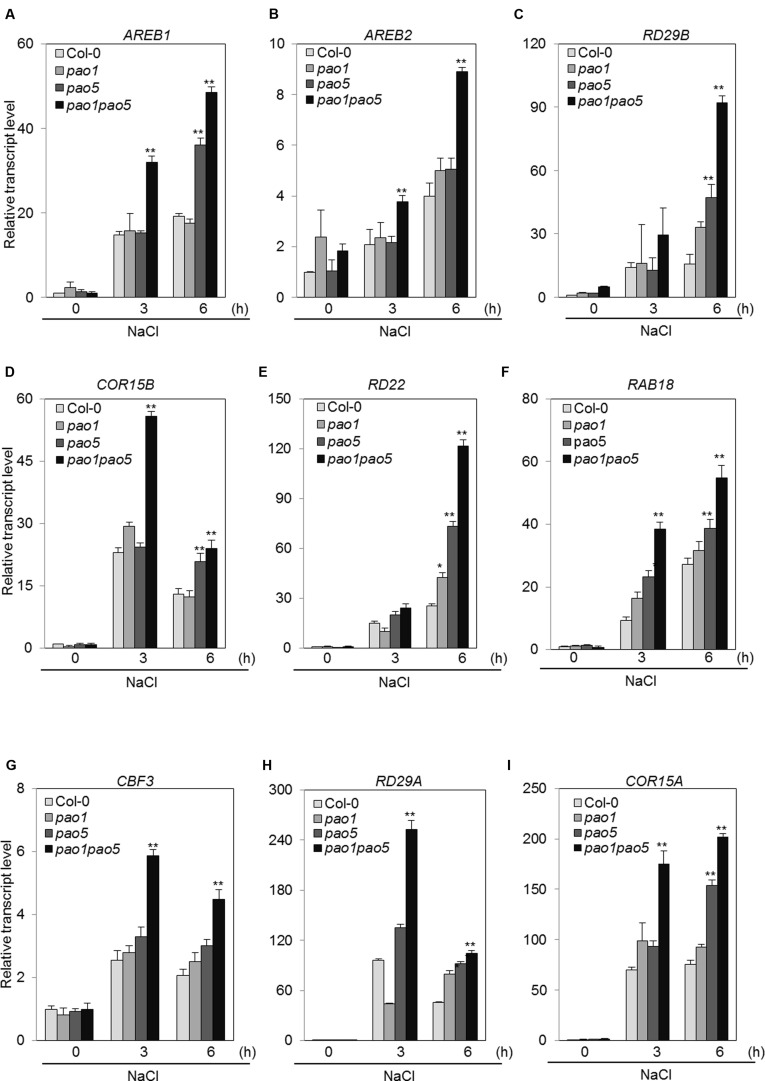
**Changes in the expression of genes involved in the ABA-dependent pathway (A–F) and in the ABA-independent signaling pathway (G–I) in WT, *pao1*, *pao5*, and *pao1 pao5* upon high salt stress.** Asterisk indicates significant difference compared to that of WT: ^∗^*P* < 0.05, ^∗∗^*P* < 0.01.

The second pathway is an ABA-dependent signaling pathway that includes several transcription factors (TFs), such as AREB1, AREB2, and the downstream component RD29B (Fujita et al., 2011). The third pathway is an ABA-independent pathway that includes several TFs, such as CBFs, and the CBFs’ target components RD29A and COR15A. Among ABA-dependent signaling pathway genes, *AREB1* transcripts accumulated 15- and 20-fold in WT at 3 and 6 h salt treatment, while they reached to 32 and 48-fold levels in the double mutant (**Figure 9A**). A similar trend was observed for the other bZIP gene, *AREB2*; i.e., it was induced to two and fourfold levels in WT at 3 and 6 h, respectively, upon NaCl treatment, whereas in the double mutant the transcript levels rose to 3.8 and 8.9-fold levels at 3 and 6 h after NaCl treatment (**Figure 9B**). Expression profiles of *RD29B*, *RD22*, and *RAB18* upon salt treatment were quite similar to that of *AREB1* (**Figures 9C,E,F**). At 6 h-salt treatment, *RD29B* transcripts increased 16-fold in WT, while in the double mutant they reached to 92-fold levels (**Figure 9C**). *RD22* and *RAB18* were up-regulated to 25 and 27-fold levels at 6 h in WT, while their transcripts reached to 120 and 54-fold levels in the double mutant (**Figures 9E,F**). *COR15B* was more quickly induced by salt treatment. In WT, the transcripts accumulated 23 and 13-fold at 3 and 6 h salt treatment, and in the double mutant they reached to 56 and 24-fold levels at 3 and 6 h treatment (**Figure 9D**). In the ABA-independent pathway, *CBF3* was induced at 2–2.6-fold levels in WT, while it was induced to 4.5–6-fold levels in the double mutant (**Figure 9G**). We tested the expression of four *CBF*s. Of them, only *CBF3* was upregulated by NaCl treatment (Supplementary Figure S7). The downstream target genes, *RD29A* and *COR15A*, of CBF3 were also induced at 45–95-fold levels and 70–75-fold levels in WT and 100–250-fold levels and 175–200-fold levels in the double mutant (**Figures 9H,I**).

## Discussion

Here we documented that the *Arabidopsis* mutant plant, *pao1 pao5*, which lost cytoplasmic PAO activity is tolerant to high salt and drought stresses through the activation of subsets of defense-related genes and the reduction of reactive oxygen species (ROS) production.

In this double mutant as well as in each single mutant, *pao1* and *pao5*, intake of Na ions was reduced to ∼75% of that in WT (**Figure 3**). The expression of *AtHKT1*, which controls Na^+^ entry into plant roots (Rus et al., 2001), was comparable among WT, *pao1, pao5* and the double mutant (**Figure 8E**), and the expression of *AtSOS1*, which encodes a Na^+^/H^+^ antiporter in plasma membrane (Zhu, 2002), was higher in both the single mutants and the double mutant in relative to that of WT (**Figure 8C**). If the respective transcript levels of the above transporter genes reflect their transporter activity, reduction of Na intake in the *pao1*, *pao5* and *pao1 pao5* is reasonable. However, the reduced Na intake does not simply explain the tolerant phenotype of the double mutant because each single mutants were salt-sensitive (Supplementary Figure S1). Two other SOS pathway members, *AtSOS2* and *AtSOS3*, were induced at higher levels in the double mutant compared to WT while their induction profiles differed (**Figures 8A,B**). Cross-talk between AtSOS2 and AtNHX1 (Apse et al., 1999; Yamaguchi et al., 2013, references therein) was evidenced (Qiu et al., 2004; Batelli et al., 2007). In *sos2* mutant, tonoplast Na^+^/H^+^ exchange activity which is performed by AtNHX1 was greatly reduced. Addition of activated SOS2 increased tonoplast Na^+^/H^+^ exchange activity in vesicles isolated from *sos2* (Qiu et al., 2004). *AtNHX1* transcripts increased to fourfold levels upon salt treatment only in *pao1 pao5* mutant but not in WT, *pao1* and *pao5* (**Figure 8D**). Since PAs can activate Ca^2+^-channels and modulate H^+^-ATPase pump activity (Pottosin et al., 2014), it is likely that AtNHX1 activity and the rate of cytoplasmic Na^+^ sequestration into the vacuole are higher in the double mutant than in the WT. Higher Na^+^ sequestration to vacuole may partially explain the salt tolerant phenomenon of the double mutant. Plants respond to high salinity by activating abscisic acid (ABA)-dependent and ABA-independent signaling pathways comprising regulatory genes like TF (Yoshida et al., 2014). Among the genes in the ABA-dependent pathway, at least two key TF genes, *AREB1* and *AREB2* (Yoshida et al., 2010), and the target genes *RD29B* and *RAB18* were significantly induced in the double mutant, and the other ABA-responsive gene *RD22*, which is regulated by RD22BP1 (MYC2; Fujita et al., 2011; Osakabe et al., 2014), was induced at the higher levels in the double mutant (**Figures 9A–C,E**). Of the ABA-independent pathway genes, *CBF3/DREB1A* and its targets, *RD29A* and *COR15A*, were also upregulated in the double mutant (**Figures 9G–I**). The enhanced expression of those defense-related genes in the double mutant may also help toward explaining why it was NaCl tolerant compared to WT, *pao1* and *pao5*.

The reason why the hyper-induction of subsets of defense genes occurs in the double mutant is still covered. AtPAO5 functions in T-Spm catabolism in *Arabidopsis* (Kim et al., 2014). In the *pao5* single mutant, T-Spm levels were twofold higher compared with those in WT (Kim et al., 2014). The recombinant AtPAO1 favored T-Spm over the other PAs (Takahashi et al., 2010). In fact, *pao1 pao5* mutant contained ∼2.8-fold higher T-Spm compared to WT (Supplementary Figure S5C). Exogenously applied T-Spm is able to induce the defense-related genes in *Arabidopsis* (Sagor et al., 2012). Therefore, endogenously accumulated T-Spm itself or a combination of T-Spm and unidentified other factor(s) may contribute as signaling molecule(s) for the gene induction. This point needs to be addressed in the future.

ROS such as H_2_O_2_ and O_2_^-^ have two ‘faces’: one is acting as toxic by-products of aerobic metabolism and the other is functioning as signaling molecules to control various processes, such as programmed cell death (PCD) and biotic/abiotic stress responses (Mittler, 2002; Miller et al., 2010). PAOs are H_2_O_2_-forming enzymes (Mittler, 2002; Cona et al., 2006; Kusano et al., 2015). Actually it was shown that ROS (H_2_O_2_) produced via a PAO action emit a signal to induce PCD in tobacco cell culture (Yoda et al., 2006). It was also reported that PAs control ROS homeostasis during salt stress in plants (Saha et al., 2015). Total PA contents in the double mutant were a bit higher (45.26 nmol/g FW), especially T-Spm, than in WT (42.81 nmol/g FW) after salt treatment (Supplementary Figure S5). Basal enzymatic activities of CAT and POX in the double mutant were ∼20–30% higher than those in WT (**Figure 6B**), whereas those of APX and SOD were comparable in WT and the double mutant (**Figure 6B**). Whether the higher CAT and POX activities contribute to the enhanced salinity tolerance of *pao1pao5* mutant is unclear. To discuss about the potency of each PA to regulate ROS homeostasis or control antioxidant enzyme activity, further works are required.

The reduced ROS, H_2_O_2_, and O_2_^-^, production in *pao1 pao5* compared with those in WT upon salt treatment was confirmed quantitatively (**Figures 4B,C**) and qualitatively (**Figure 5**). Intriguingly histochemical staining method showed that net production of H_2_O_2_ and O_2_^-^ was clearly lower in the double mutant compared with those in WT, *pao1, pao5* (**Figure 5**) and in the other double mutant *pao2 pao4* (Supplementary Figure S6). This reduced ROS production may partially explain the different response against salt stress among WT, each single mutants and *pao1 pao5* mutant.

Lastly, in keeping with this topic, we have to refer the works done by Moschou et al. (2008a): they generated transgenic tobacco plants overexpressing maize-derived *PAO*. Those transgenic plants were unable to cope with oxidative stresses induced by methyl viologen, menadione, and exogenous H_2_O_2_ (Moschou et al., 2008a). Then, they generated transgenic tobacco plants in which the expression of the apoplastic PAO gene was downregulated by an anti-sense method: those plants exhibited increased biomass production on MS medium supplemented with 100 mM or 200 mM NaCl (Moschou et al., 2008b). They interpreted that, upon salt stress, plants secreted Spd into apoplastic space, which was catabolized by apoplastic PAO and produced H_2_O_2_. The anti-sense PAO plant produced less H_2_O_2_ and exhibited less PCD, on the other hand, the tobacco plant overexpressing the apoplastic PAO gene produced higher levels of H_2_O_2_ but failed to induce stress-responsive genes.

In *Arabidopsis*, there are five *AtPAO* genes. Of them, *AtPAO1*, *AtPAO2*, and *AtPAO3* were responsive to salt stress, whereas *AtPAO4* and *AtPAO5* were not (Supplementary Figure S8). *AtPAO1* is expressed in the transition region between the meristematic and the elongation zone of roots and anther tapetum. Its expression is also ABA responsive, especially in root tip (Fincato et al., 2012). Especially *AtPAO1* seemed to be involved in salt stress response. Since the *pao1 pao5* mutant completes its life cycle, the loss of cytoplasmic PAO activity does not disturb full development. It is of interest whether other plants silencing cytoplasmic PAO pathway become salt and/or drought tolerant. If it is the case, it provides an alternative way to generate salt/drought tolerant plants.

## Author Contributions

GS, SZ, SK, TB, and TK designed the experiments. GS, SZ, SK, SS and TB performed the experiments. GS, SZ, SK, SS analyzed the data. TB and TK wrote the paper.

## Conflict of Interest Statement

The authors declare that the research was conducted in the absence of any commercial or financial relationships that could be construed as a potential conflict of interest.
