# Supplementary material for: Reducing Cytoplasmic Polyamine Oxidase Activity in Arabidopsis Increases Salt and Drought Tolerance by Reducing Reactive Oxygen Species Production and Increasing Defense Gene Expression
Source: Front Plant Sci. 2016 Feb 29;7:214. doi: 10.3389/fpls.2016.00214 (PMC4770033; doi:10.3389/fpls.2016.00214)
Supplement: Supplementary file 1 [file Data_Sheet_1.PDF]

# *Supplementary Material*

**Reducing cytoplasmic polyamine oxidase activity in Arabidopsis increases salt and drought tolerance by reducing reactive oxygen species production and increasing defense gene expression**

G.H.M. Sagor[1], SiyuanZhang[1], Seiji Kojima[1,2], Stefan Simm[3], Thomas Berberich[4], Tomonobu Kusano[1]

*** Correspondence:** Tomonobu Kusano, Tohoku University, Graduate School of Life Sciences, 2-1-1 Katahira, Aoba, Sendai, Miyagi 980-8577, Japan

Tel & Fax: +81-22-217-5709; E-mail:kusano@ige.tohoku.ac.jp

Supplementary Data

Supplementary Material should be uploaded separately on submission. Please include any supplementary data, figures and/or tables.

Supplementary material is not typeset so please ensure that all information is clearly presented, the appropriate caption is included in the file and not in the manuscript, and that the style conforms to the rest of the article.

## 1 Supplementary Figures and Tables

There are five supplementary figures and one supplementary table.

### 1.1 Supplementary Figures

**Figure S1:Growth phenotypes of the five *pao* mutants in the presence of 100 mMNaCl.** Sterilized seeds of WT, *pao1*, *pao2*, *pao3*, *pao4*, and *pao5* were placed onto half-strength MS agar medium or half-strength MS agar medium containing 100 mMNaCl and incubated for 14 days in a vertical position. **A,** Growth phenotypes of salt-treated Arabidopsis *pao* mutant plants. The representative growth phenotypes were photographed. **B,** Comparison of primary root lengths. The values indicate mean ± SE (*n* = 5).

**Figure S2:Schematic illustration of the T-DNA insertion sites in *AtPAO1*, *AtPAO2*, *AtPAO4*,and *AtPAO5* (A),and confirmation of the T-DNA insertions in both DNA strands of *pao1pao5* and *pao2pao4* by genomic DNA-PCR (B, C).**

**Figure S3.Relative transcript levels of *PAO1* to *PAO5* in WT and the two double mutants, *pao1 pao5* and *pao2 pao4*;** qRT-PCR was performed as described by Kim et al. (2014).

**Figure S4**. **Drought-tolerance phenotype of *pao1 pao5* mutant.** Seven-day-old plantlets (5 each) of WT (Col-0), *pao1*, *pao5* and *pao1 pao5* were transferred onto the pots. After growing for 12 days under well-watered condition, the plants were subjected to drought stress (stopping water supply) for next 40 days.

**Figure S5.Changes in PA contents of WT and *pao1pao5* seedlings during 100 mMNaCl treatment.A,** Put; **B,**Spd; **C,** T-Spm; **D,**Spm. Asterisk indicates significant difference compared to the values of non-stressed condition unless indicated:$^{*}P<0.05$; $^{**}P<0.01$.

**Figure S6. Histochemical analysis of $O_2^-$ (A, C) and $H_2O_2$ (B, D) accumulation in WT and *pao2pao4* double mutant after NaCl treatment.**(A, C).NBT staining.(B, D).DAB staining. (A, B) Stained whole seedling; (C, D) enlarged cotyledon leaves. Bar indicates 1 mm.

**Figure S7**. **Expressional change of *CBF1, CBF2, CBF3* and *CBF4* in Arabidopsis WT seedlings treated with 100 mM NaCl for certain time periods.** Grey bar and black bar indicate WT (Col-0) and *pao1 pao5* mutant, respectively.

**Figure S8**. **Expressional change of *AtPAO*s upon 100 mM NaCl treatment.** Two-week-old Arabidopsis seedlings were transferred onto 1/2 MS solution and incubated for 24 h under continuous light condition at 22°C, then one half of the plant samples were treated by 100 mM NaCl, and the other half of the samples were left un-treated (Control). At 0 h, 3 h and 6 h, the plants were harvested.

**Supplemental Data**

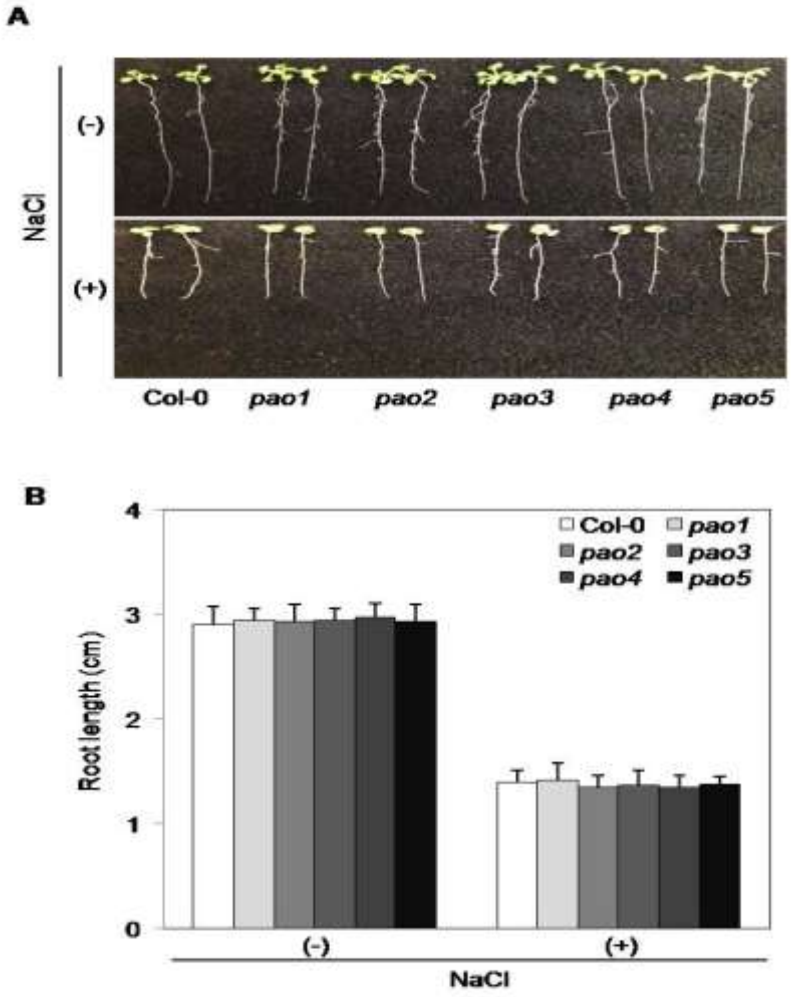


**Supplementary Figure 1.**Growth phenotypes of the five *pao* mutants in the presence of 100 mM NaCl. Sterilized seeds of WT, *pao1*, *pao2*, *pao3*, *pao4*, and *pao5* were placed onto half-strength MS agar medium or half-strength MS agar medium containing 100 mM NaCl and incubated for 14 days in a vertical position. **A**. Growth phenotypes of salt-treated Arabidopsis *pao* mutant plants. The representative growth phenotypes were photographed. **B**. Comparison of primary root lengths. The values indicate mean± SE ($n$ = 5).

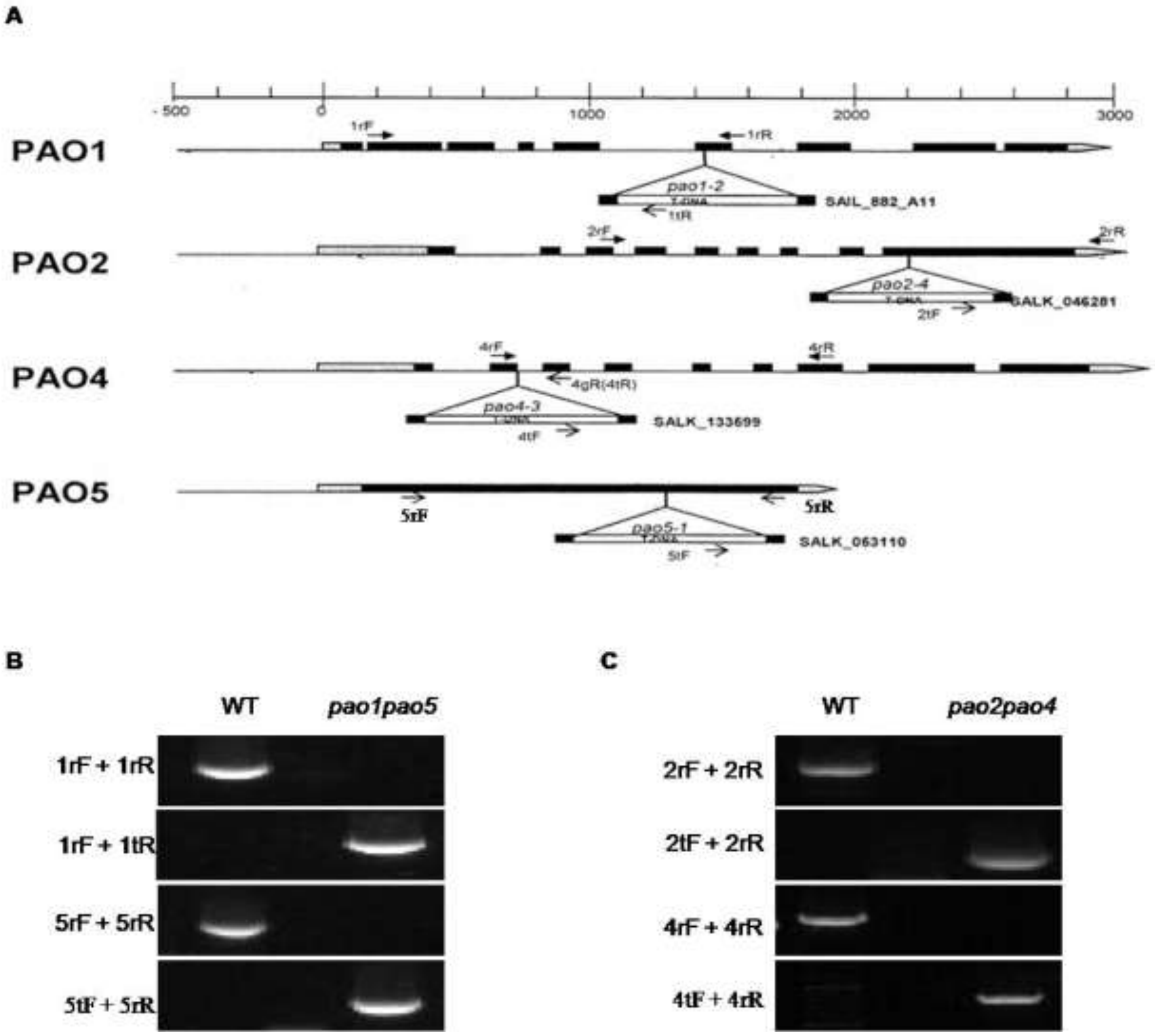


**Supplementary Figure 2.** Schematic illustration of the T-DNA insertion sites in *AtPAO1, AtPAO2, AtPAO4*,and *AtPAO5* (**A**), and confirmation of the T-DNA insertionsin both DNA strands of *pao1pao5* and *pao2pao4* by genomic DNA-PCR (**B**, **C**).

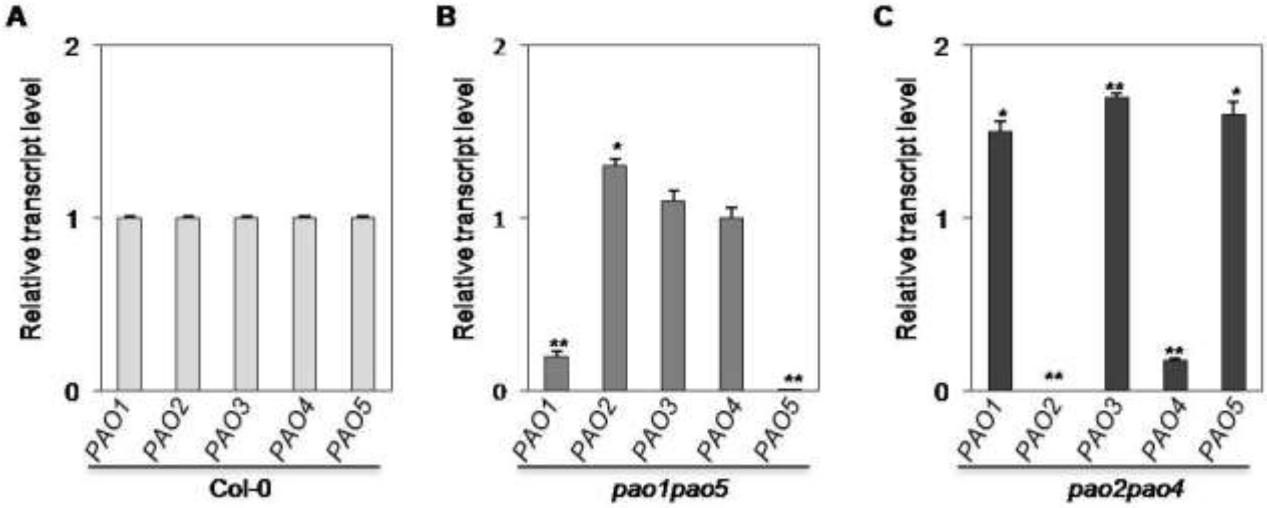


**Supplementary Figure 3.**Relative transcript levels of *PAO1* to *PAO5* in WT (Col-0) and the two double mutants, *pao1 pao5* and *pao2 pao4*; qRT-PCR was performed as described by Kim et al. (2014). The each *PAO* transcript level in WT was set as 1.0 and the relative transcript levels were shown. Asterisk indicates the significant difference compared to that of WT: $^{*}P<0.05$, $^{**}P<0.01$.

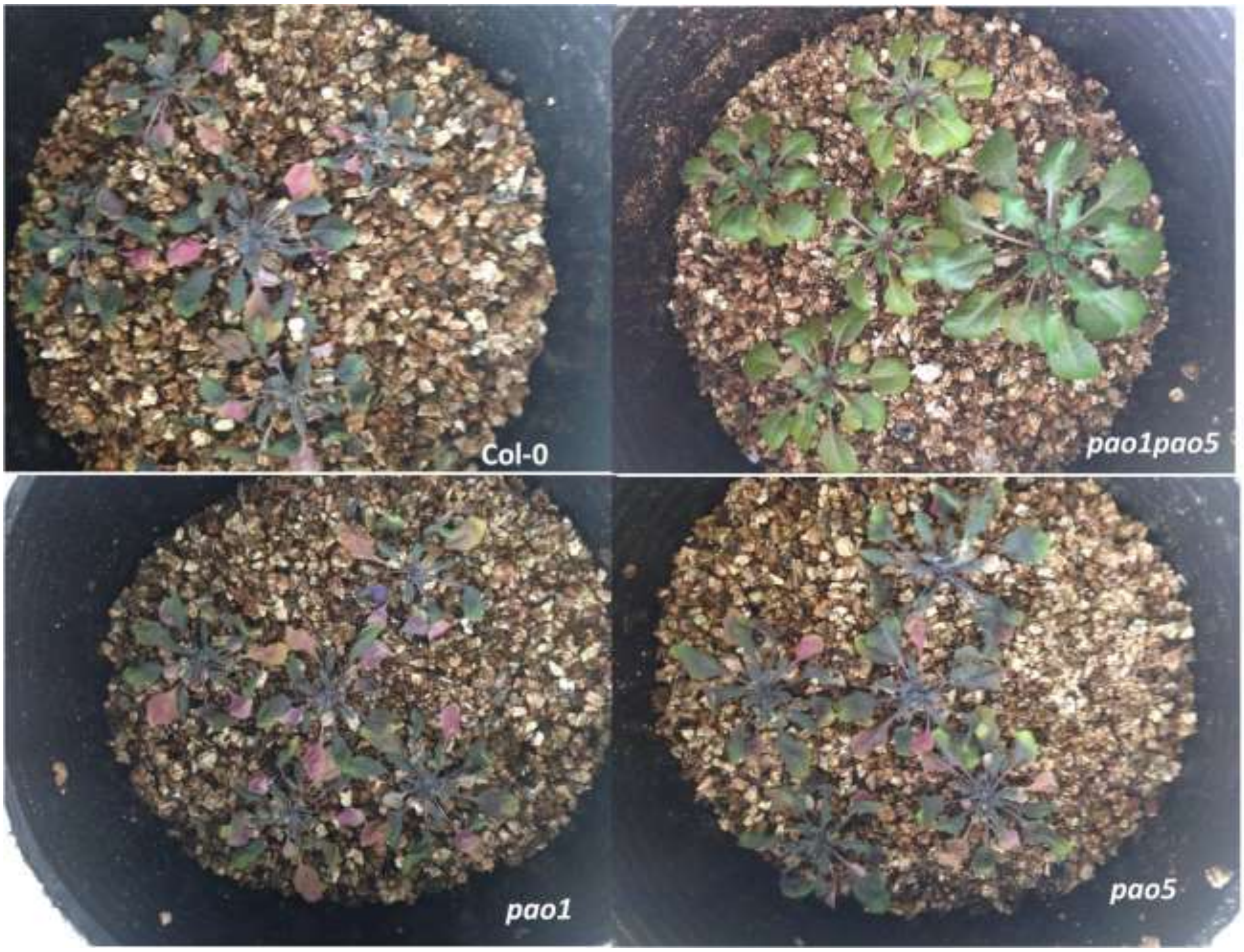


**Supplementary Figure 4. Drought-tolerance phenotype of *pao1 pao5* mutant.** Seven-day-old plantlets (5 each) of WT (Col-0), *pao1*, *pao5* and *pao1 pao5* were transferred onto the pots containing the mixture soil. After growing for 12 days under well-watered condition, the plants were subjected to drought stress (stopping water supply) for next 40 days.

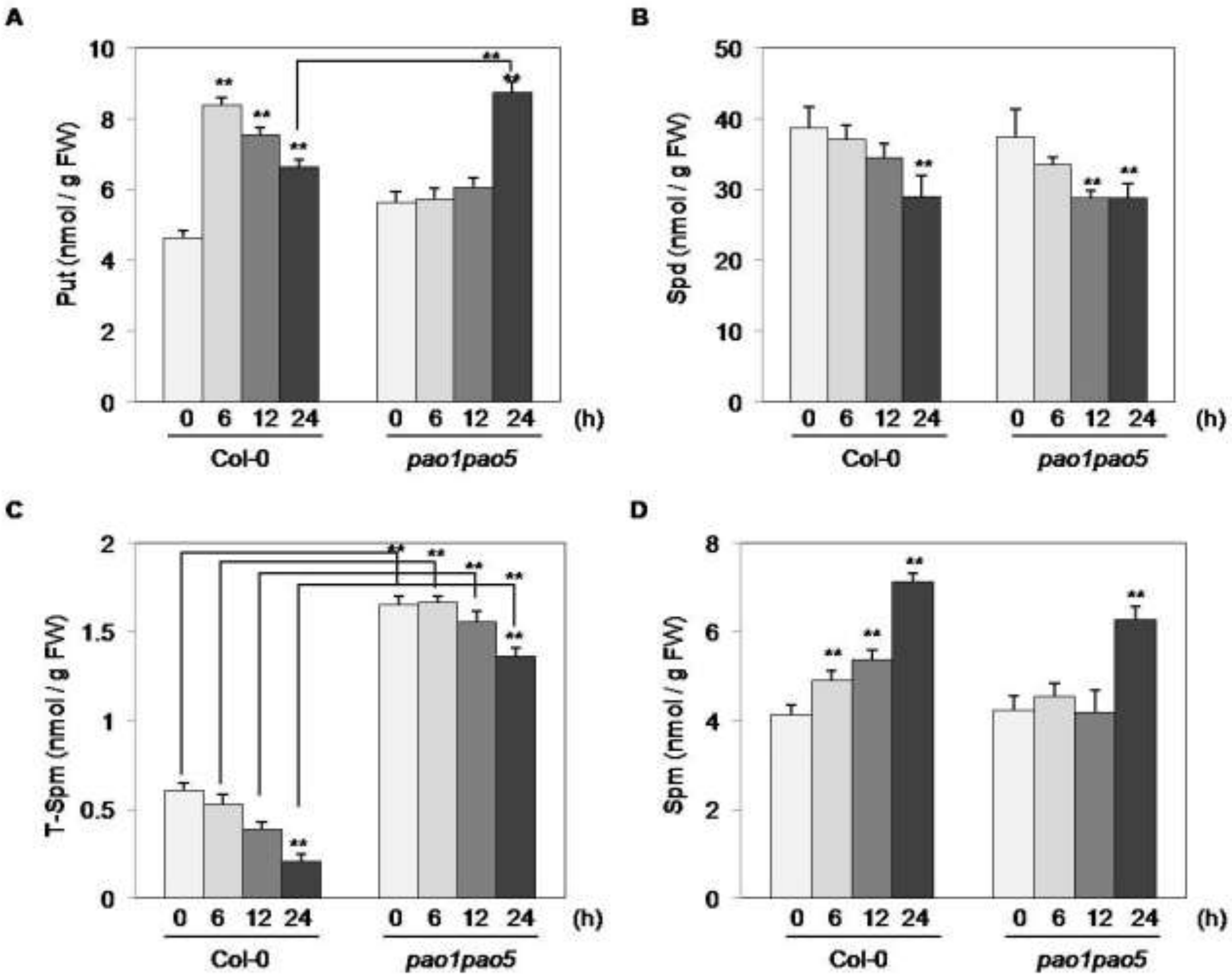


**Supplementary Figure 5.** Changes in PA contents of WT and *pao1pao5* seedlings during 100 mM NaCl treatment.**A**, Put; **B**, Spd; **C**, T-Spm; **D**, Spm. Asterisk indicates significant difference: $^{*}P<0.05$; $^{**}P<0.01$.

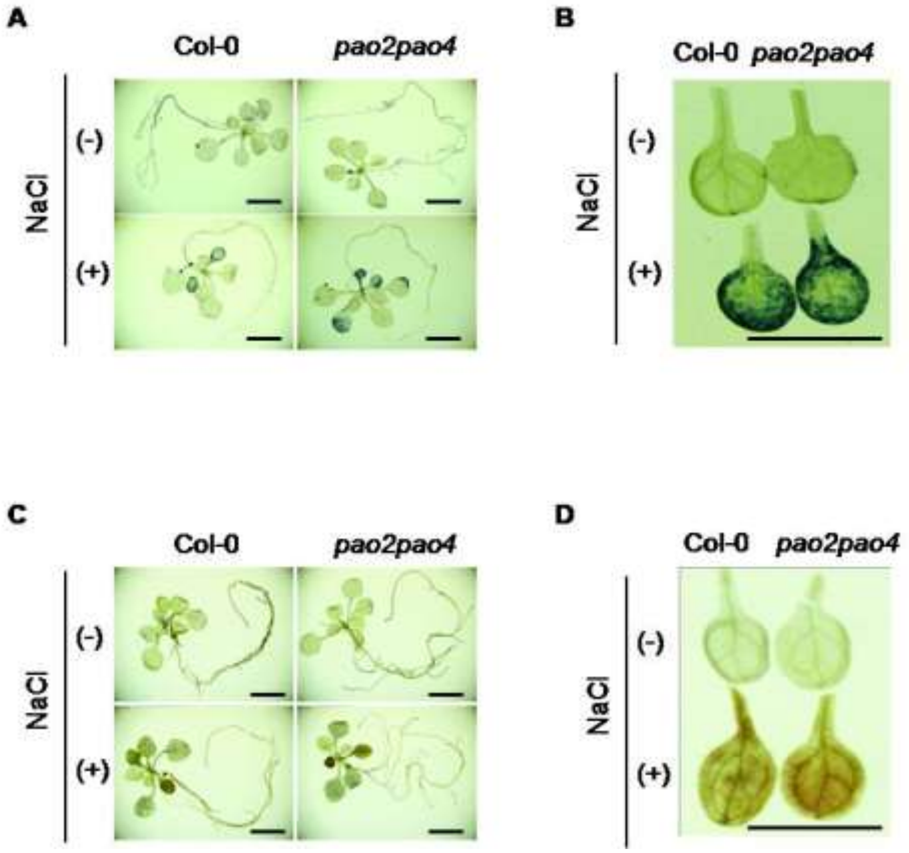


**Supplementary Figure 6.** Histochemical analysis of $O_2^-$ (**A, C**) and $H_2O_2$ (**B**, **D**) accumulation in WT and *pao2pao4* double mutant after NaCl treatment.(**A, C**).NBT staining. (**B, D**).DAB staining. (**A, B**) Stained whole seedling; (**C, D**) enlarged cotyledon leaves. Bar indicates 1mm.

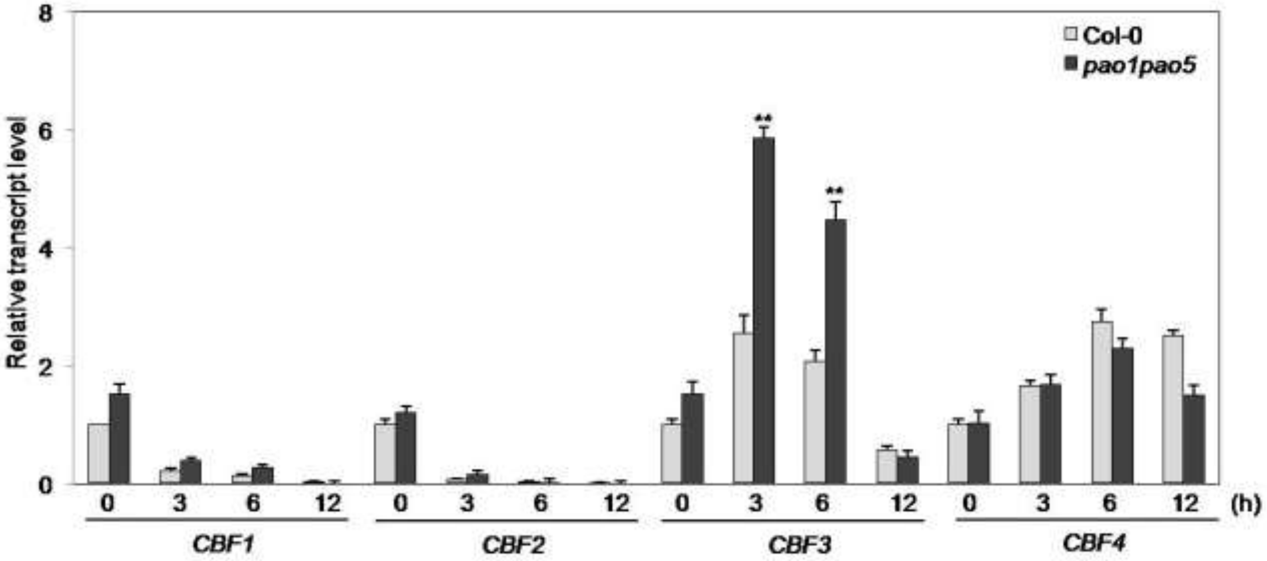


**Supplementary Figure 7.** Expressional change of *CBF1*, *CBF2*, *CBF3* and *CBF4* in Arabidopsis WT seedlings treated with 100 mM NaCl for certain time periods. Grey bar and black bar indicate WT (Col-0) and *pao1 pao5* mutant, respectively.

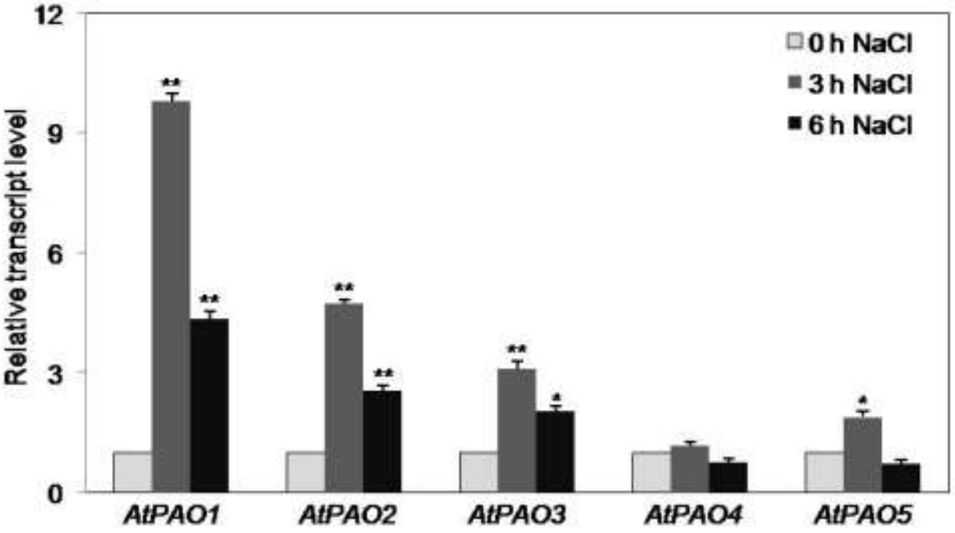


**Supplementary Figure 8.** Expressional change of Arabidopsis *PAO* genes in response to 100 mM NaCl. Two-week-old Arabidopsis WT seedlings were transferred onto 1/2 MS solution and incubated for 24 h under continuous light condition at 22°C, then one half of the plant samples were treated by 100 mM NaCl, and the other half of the sample were left un-treated. At 0 h, 3 h and 6 h, the plants were harvested.

## 1.2 Supplementary Tables

**Supplementary Table 1.Primers used in RT-PCR analysis.**

| Experiment | Name | Sequence(5' -3') |
|---|---|---|
| RT-PCR | AtPAO1_rF | GATACTGGAAGCGACGGATCGGAT |
| | AtPAO1_rR | TTCGTATACGGAACCATCCTCTGT |
| | AtPAO1_tR | TAGCATCTGAATTTCATAACCAATCTCGAT |
| | AtPAO5_rF | GCCTTCCATTGTTGAATCCATCTCTG |
| | AtPAO5_rR | AGGCACCATGAGTTGTGGAGTAATG |
| | AtPAO5_tF | TGGTTCACGTAGTGGGCCATCG |
| | AtPAO2_rF | AAGGTCGCCCTCCGTGATTGTGATCG |
| | AtPAO2_rR | GTACTTGAATTCTTAAAGATGATTATCATA |
| | AtPAO2_tR | GTACTTGAATTCTTAAAGATGATTATCATA |
| | AtPAO4_rF | TGTGCAGCCTTCTGTTATTGTGATTG |
| | AtPAO4_rR | ATCAAGATCTTTTGCAATTGTTCT |
| | AtPAO4_tR | TATGGATACGACCACCAATCCTATCGCGTC |

**Supplementary Table 2.Primers used in qRT-PCR analysis.**

| Experiment | Name | Sequence(5' -3') | Amplicon length (bp) |
|---|---|---|---|
| qRT-PCR | AtPAO1_F | TGCCGGTGTCGGTGGTA | 60 |
| | AtPAO1_R | CGTTAAAACGCGAGGCAAGT | |
| | AtPAO2_F | GATCCTTTGGCCCCCATT | 58 |
| | AtPAO2_R | TACGACGATCGGCGATATCTG | |
| | AtPAO3_F | CCGTTCATTGTTGAATCATGGA | 63 |
| | AtPAO3_R | AATATCTTTACGCAGCTGACGAT | |
| | AtPAO4_F | CGCGAACGAAGCCGTTATT | 79 |
| | AtPAO4_R | CAATGGCGAGGATTCAAAAAC | |
| | AtPAO5_F | CCGGGAGGATTCCGAGTT | 63 |
| | AtPAO5_R | ATGGTCGCGGTTCTTCTCAT | |
| | AtCBP20_F | GGCTCATTGGGAGCTTATCCT | 60 |
| | AtCBP20_R | CCACGCCTTCCATTTCCATA | |
| | AtAREB1_F | GGTGGTCTTGTGGGACTTGGA | 354 |
| | AtAREB1_R | CTTCAAGCTCCACGGTGTAAG | |
| | AtAREB2_F | GGGTTTTAGGGCTTGGATGCT | 919 |
| | AtAREB2_R | TTCACAGGCGCAGAAAATGCT | |
| | AtCOR15A_F | CACAGCGGAGCCAAGCAGAG | 76 |
| | AtCOR15A_R | GTTGAGAAACGACGACGAACTGAG | |
| | AtCOR15B_F | GATGACCTCAACGAAGCCACAAAG | 338 |
| | AtCOR15B_R | TTTCTCGCCATCCGCCAAGG | |
| | AtCBF1_F | GCTACGAATCCCGGAGTCAA | 63 |
| | AtCBF1_R | CAGCAGCCGCTTTTTGGA | |
| | AtCBF2_F | GCTCTCCGTGGCAGATCTG | 59 |
| | AtCBF2_R | CCGCCAAGCCGAGTCA | |
| | AtCBF3_F | GCGGCGGCTGAAGCT | 52 |
| | AtCBF3_R | TCGTCGCATCACACATCTCA | |
| | AtCBF4_F | TTGGCGGCTTCGTATTCCT | 57 |
| | AtCBF4_R | AGCTTCAGACGCAGCTTTCTG | |
| | AtRD29A_F | GTGCGACGGAGGAGGTGAAG | 79 |
| | AtRD29A_R | GGAACAACAGTGGAGCCAAGTG | |
| | AtRD29B_F | GCGGAGGGGAAAGGACATGGTGAGGCG | 300 |
| | AtRD29B_R | GGTTCACAAACAAACAGAGGCATCATAC | |
| | AtRD22_F | GCCAGGGACCGTTCCGGTCTGCCACTTCC | 309 |
| | AtRD22_R | CCGAAATGGTAACATTTCAGCTCTAATTT | |
| | AtRAB18_F | GAGGGAGGAGGAAGAAGGGAATAAC | 88 |
| | AtRAB18_R | ACCGTAGCCACCAGCATCATATC | |
| | AtSOS1_F | AAACCCGTAAACATGCGTGTC | 63 |
| | AtSOS1_R | GCAGAATTGCATATACATTC | |
| | AtSOS2_F | GGTTTCGGATTTCGGACTCA | 65 |
| | AtSOS2_R | CACATGTGGTACGCAGAAGTTCTA | |
| | AtSOS3_F | TGGAGTGATCGAGTTTGGTGAA | 60 |
| | AtSOS3_R | GCGCGCTTGGATGGAA | |
| | AtNHX1_F | CCCACTCGAACCGTGCAT | 57 |
| | AtNHX1_R | GGGTCGCATGAAGGAGTCA | |
| | AtHKT1_F | TGGTTTCACTACCGGGTACA | 311 |
| | AtHKT1_R | CGAGGATTAACGATGATGCAA | |
| | AtCAT1_F | TCGATCCGCTTGATGTCACA | 62 |
| | AtCAT1_R | ACCAACAGGTTGTAAAGGCAAGA | |
| | AtCAT2_F | CTGCTTCAAACCCGTGTCTTC | 62 |
| | AtCAT2_R | TAGTTTGGTCCAAGACGGTGTCT | |
| | AtCAT3_F | TCCGCTGCGCTGAGAAA | 62 |

| | | |
|---|---|---|
| AtCAT3_R | GACCTTTGTTCGAATTCCAGTGT | |
| AtAPX1_F | GCCCACACTCTGGGACGAT | 59 |
| AtAPX1_R | GATGTCCATGCACCTTCGAA | |
| AtAPX2_F | TGGGTCGGTGCCACAAG | 59 |
| AtAPX2_R | GAGCGGGTTTGGTGTCCAT | |
| AtAPX3_F | CAAACTCCTCAGCAGGCAAAG | 59 |
| AtAPX3_R | GAACGCACTCTGTGCCAGAA | |
| AtAPX4_F | CCGCCGTGATGTTCTCAAA | 62 |
| AtAPX4_R | AACCGTTGCCTATTAATTCCATTC | |
| AtSAPX_F | AGTTGCATTATCTGGTGCTCACA | 67 |
| AtSAPX_R | AGGCTTCCCCCAACCACTA | |
| AtSOD1_F (Fe) | CAAGTGCTGTCACCGCAAAC | 56 |
| AtSOD1_R(Fe) | AGCATCCAGTGCGAATGGA | |
| AtSOD2_F (Fe) | TGCTCTGGAACCGCATATGA | 58 |
| AtSOD2_R(Fe) | GTGATGTTTGCCCCAGTGATAA | |
| AtSOD3_F (Fe) | TCTGGGAGTCGATGCAACCT | 60 |
| AtSOD3_R(Fe) | TCTGCTCAAGAACACCCTTTTG | |
| AtSOD_F (Mn) | GGGAGATGCTTCCACTGTTGTT | 57 |
| AtSOD_R (Mn) | CCTCCGCCGTTGAATTTG | |
| AtSOD1_F (Cu / Zn) | TCAACCCCGATGGTAAAACAC | 58 |
| AtSOD1_R(Cu / Zn) | TCACCAGCATGTCGATTAGCA | |
| AtSOD2_F (Cu / Zn) | CGCGGCGTCTCTCTCAA | 59 |
| AtSOD2_R(Cu / Zn) | ACGGCGAAGGAAACAGATTG | |
| AtSOD3_F (Cu / Zn) | CGGGAGGGCGGTTGTT | 52 |
| AtSOD3_R(Cu / Zn) | CCCTCCTTTCCCAAGGTCAT | |
